# Neuromodulation and Individuality

**DOI:** 10.3389/fnbeh.2021.777873

**Published:** 2021-11-24

**Authors:** Ryan T. Maloney

**Affiliations:** Department of Organismic and Evolutionary Biology, Harvard University, Cambridge, MA, United States

**Keywords:** individuality, neuromodulation, animal personality, variability, bet-hedging, robustness, plasticity

## Abstract

Within populations, individuals show a variety of behavioral preferences, even in the absence of genetic or environmental variability. Neuromodulators affect these idiosyncratic preferences in a wide range of systems, however, the mechanism(s) by which they do so is unclear. I review the evidence supporting three broad mechanisms by which neuromodulators might affect variability in idiosyncratic behavioral preference: by being a source of variability directly upstream of behavior, by affecting the behavioral output of a circuit in a way that masks or accentuates underlying variability in that circuit, and by driving plasticity in circuits leading to either homeostatic convergence toward a given behavior or divergence from a developmental setpoint. I find evidence for each of these mechanisms and propose future directions to further understand the complex interplay between individual variability and neuromodulators.

## Introduction

Across a wide range of species, from *C. elegans* (Stern et al., 2017) to humans (Sanchez-Roige et al., 2018), individuals exhibit idiosyncratic behavioral preferences, even when they are genetically identical and raised in similar environments. These differences seem to arise due to underlying stochastic processes during development, causing the same genetic profile to lead to a range of neural phenotypes.

These stochastic processes play important roles in development, however, how they relate to variation in behavior is not always clear. Stochastic processes in development may resolve to highly stereotyped results as well as variable ones, depending on mechanisms to induce robustness (Johnston and Desplan, 2010). Similarly circuits with differing numbers of neurons, connections, and ion conductance can converge on seemingly identical behaviors (Prinz et al., 2004; Daur et al., 2012; Goaillard and Marder, 2021). Understanding when and how variations in the underlying circuit lead to divergence in behavior is crucial to understanding the developmental and ecological context of individuality, defined here as biases or preferences in an individual that differentiate it from other animals in a population.

Individuality plays an important role in the survival of a species (Cohen, 1966; Hopper, 1999). Divergent preferences among a species allow the species to hedge against unpredictable environments by having a range of phenotypes adapted to different possible environments, ensuring some proportion of the population survives regardless of environmental fluctuations (Kain et al., 2015; Xue et al., 2019). The degree to which individuals within an isogenic population show divergent preferences is strongly influenced by genetics, as shown by studies showing differing amounts of individuality between isogenic populations with different genetic backgrounds (Ayroles et al., 2015; Bruijning et al., 2020), demonstrating that intra-genotypic variability is under evolutionary control. This is supported by observed differences in population variability that match theoretical predictions of environments where variability provides a fitness advantage (Akhund-Zade et al., 2020; Krams et al., 2021). One key proposed mechanism for the regulation of individuality is neuromodulation.

Neuromodulators play a key role in regulating behavior at multiple scales. Neuromodulators are a diverse set of chemicals with a wide range of receptors, kinetics, targets, and roles, however, they have several broadly shared characteristics. Compared to conventional neurotransmitters, neuromodulators are characterized by volume release, broad connectivity, and slower and longer kinetics (Bargmann and Marder, 2013). Because of their ability to trigger widespread shifts in network function across the nervous system, changes in neuromodulation can trigger large shifts in behavior (Lee and Dan, 2012; Taghert and Nitabach, 2012). Within an individual, these shifts allow organisms to adjust their behavior based on context, such as in response to satiety (Marella et al., 2012), social conflict (Rittschof et al., 2014), arousal (Arnsten et al., 2012), experiences (Likhtik and Johansen, 2019) circadian rhythm (Witkovsky, 2004) or stress (Rodrigues et al., 2009). Within eusocial insects, neuromodulators can drive differences in behavior between sub-castes (Kamhi et al., 2015), and help regulate group behavior in response to environmental cues (Kamhi et al., 2017). Neuromodulators and hormones have also been proposed to serve as loci for evolutionary shifts in behavior based on their broad targets affecting a variety of disparate traits, making it easier to coordinate shifts in multiple traits to linked to advantageous behavioral shifts (Cox et al., 2016; Garland et al., 2016). Key to neuromodulators’ role in the evolution of behavior is the ability for small shifts in expression levels and localization of elements of the neuromodulatory systems to shift behavior (Katz and Lillvis, 2014), avoiding the need to create *de novo* behaviors and circuits to change behavior in response to evolutionary pressure. Artificial selection experiments have shown that selection pressure can act via changes in neuromodulator levels to drive rapid changes in behavior (Pantoja et al., 2020). Similarly, neuromodulatory systems may serve as loci for individuality—sites where idiosyncratic circuit differences cause idiosyncratic behavior differences (Skutt-Kakaria et al., 2019). Neuromodulatory systems are prime targets to be loci for individuality based on their ability to provide coordinated shifts in function over multiple circuits in the nervous system, and therefore enable coordinated changes in behavior with comparatively few points of variation. A wide range of studies across different behaviors and species have shown that changes in neuromodulators can affect the manifestation of individuality (Table 1), suggesting that neuromodulators may play a key (though not exclusive) role in driving individuality among populations.

**TABLE 1:** Examples of ties between neuromodulators and individuality.

***Study***	**Species**	**Population type**	**Neuromodulators studied**	**Output studied**	**Effect on variability in output**	**Correlation**
Stern et al., 2017	*C. elegans*	Isogenic, backcrossed mutants	Serotonin	Roaming Fraction	Decreased Serotonin leads to decreased persistence in preference	Positive
			Tyramine, octopamine, npr-1, daf-7	Roaming speed	Decreased Neuromodulator increases bias toward high or low speeds	Negative
Omura et al., 2012		Isogenic	Dopamine	Roaming Speed	Decreased dopamine decreases variability	Negative
Pantoja et al., 2016	*D. rerio*	Outbred	Serotonin	Acoustic Startle Response Habituation	Decreased serotonin increase habituation	Negative
Kain et al., 2012	*D. melanogaster*	Isogenic	Serotonin	Phototactic Preference	Decreased Serotonin increases population variability	Negative
Honegger et al., 2019	*D. melanogaster*	Isogenic	Serotonin	Olfactory Preference	Decreased Serotonin decreases population variability	Positive
			Dopamine	Olfactory Preference	Increased Dopamine increases population variability	Positive
Krams et al., 2021	*D. melanogaster*	Wild Caught sibling populations from multiple locations	Serotonin	Phototactic Preference	Decreased Serotonin increases population variability	Negative

Despite this clear evidence that neuromodulators play an important role in regulating variation in behavioral preferences in many systems, the mechanisms by which they do so are unclear due to a combination of limited study and the complexity and heterogeneity of neuromodulators. Below, I describe three broad categories by which neuromodulators might affect individuality: variability in neuromodulation, altering circuit function to mask or accentuate circuit variability, and driving plasticity in the underlying circuit. Each of these categories of mechanisms provides different experimental predictions about how neuromodulation affects behavioral individuality, providing an opportunity to deepen our understandings of the myriad of ways neuromodulators might influence individuality in different systems and behaviors.

## Variation in Neuromodulators as a Driver of Individuality

**FIGURE 1:**
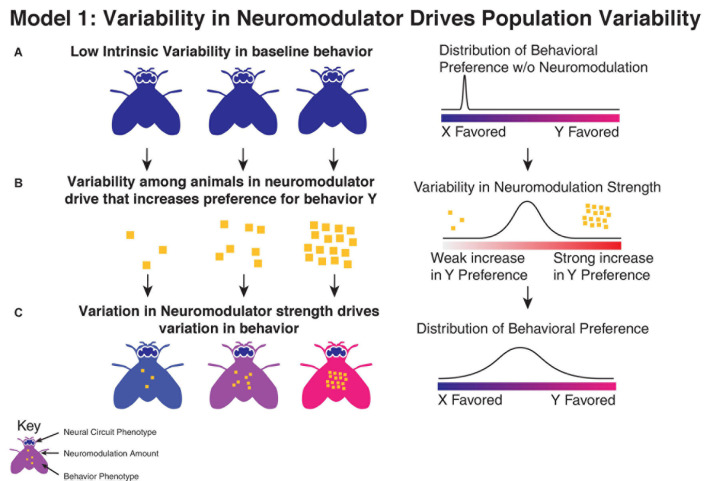
Variation in neuromodulation as driver of behavioral output. **(A)** In this model, individuals show limited variability in their behavior (indicated by individual body color) in the absence of neuromodulation, reflecting low variability in underlying parameters in neural circuits (indicated by brain coloring). **(B)** Individuals instead show significant variability in the strength of their neuromodulatory drive, which drives changes in their behavior. **(C)** This leads to increased variation in the observed behavioral preference.

One potential mechanism through which neuromodulators may drive individuality is by being themselves variable between individuals (Figure 1). Neuromodulators have strong effects on behaviors, and within an animal shifts in neuromodulators are a driver of trial to trial variability (McCormick et al., 2020). Variation in the amount of neuromodulation, via differences in receptor expression, production of neuromodulators, or activity in neuromodulatory neurons, could drive differences in behavioral preference between individuals. Among genetically diverse populations, variations in the activity of neuromodulatory neurons or mutations in receptors can manifest changes in personality (Sanchez-Roige et al., 2018). Outbred zebrafish populations show significant variation in acoustic startle response that correlate with the physiology of neuromodulatory dorsal raphe neurons (Pantoja et al., 2016), with individuals showing a higher fraction of serotonergic dorsal raphe nucleus neurons active during escape attempts also showing a decreased habituation to startle. Epigenetic changes in expression of neuromodulatory components have also been tied to differences in personality (Cardoso et al., 2015; Puglia et al., 2018; Park et al., 2020). In addition to changes in the global levels of neuromodulation, behavioral variation could also be due to variation in the targets of neuromodulatory neurons, such as has been observed in *C. elegans*, where electron microscopy reveals that neuromodulatory neurons show higher synapse count variation than conventional neurons (Witvliet et al., 2021).

Variation in neuromodulation is limited in its ability to explain all individuality, however. In cases where silencing a neuromodulator leads to an increase in variability, it suggests that the root cause of the behavioral variability is a source other than variability in the direct effect of the neuromodulator in question.

Furthermore, it is difficult to reconcile this explanation with cases where individuality appears to be driven by the asymmetric innervation of known non-modulatory cell types, for example variation in object orientation in *Drosophila melanogaster* is driven by asymmetries in DCN neurons (Linneweber et al., 2020). In these cases, direct variation in neuromodulators cannot account for the observed variability.

## Neuromodulators as Shapers of the Relationship Between Circuits and Behavior

In contrast to conventional neurotransmitters, neuromodulators are frequently insufficient to directly drive activity in neurons, instead altering intrinsic properties of the neuron and filtering the response to conventional neurotransmitters. Because the activity of neurons and circuits is non-linear and based on a wide range of factors, changes in intrinsic properties due to neuromodulation can either lead to a regime where a large variance in a parameter has little or no effect on the output of a circuit or a regime where small changes lead to large changes in behavior (Goldman et al., 2001; Grashow et al., 2009; Hamood and Marder, 2014; Marder et al., 2014). This observation mirrors similar observations and theory in evolutionary genetics, where certain mutations lead to canalization, suppressing phenotypic variations despite underlying variability in the genes (Félix and Barkoulas, 2015).

**FIGURE 2:**
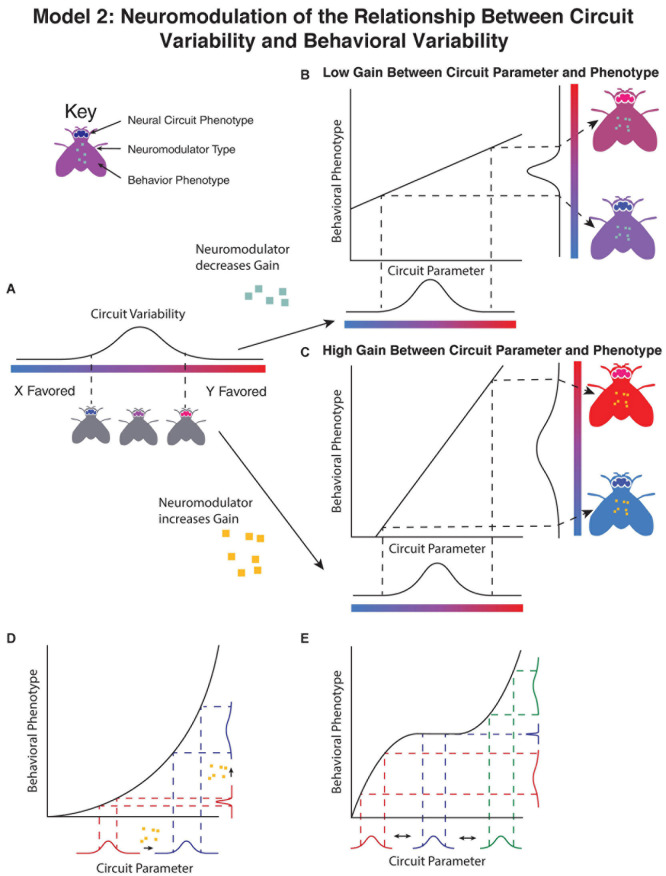
Neuromodulators adjust the relationship between underlying circuit variability and observed variation in behavior. **(A)** In this model, the non-modulatory parameters of the circuit lead to a distribution of behavioral phenotypes in the absence of modulatory input. The addition of neuromodulators either decrease the slope of the relationship between the circuit parameter and behavioral phenotype **(B)**, leading to a decrease in the observed variability, or increase the slope of the relationship between the underlying circuit and the behavioral phenotype **(C)**, increasing the variability in behavior. The change in gain can occur either through by directly altering the relationship, or by altering the mean of a parameter upstream of a non-linear relationship **(D)**. In this example a neuromodulator shifts the mean value of a circuit parameter without altering its variance, however, due to the non-linear relationship between the circuit parameter and the observed behavioral phenotype, the variance of the behavioral phenotype is changed. **(E)** Shifts in parameters by neuromodulators may have inconsistent effects on the variability of observed behavior based on the starting value of the parameter and the relationship between circuit parameter and behavioral phenotype. In this example, variation in a circuit parameter (blue) under some conditions leads to no variation in the observed behavior, however, shifting the mean in either direction increases the variability in the observed behavioral phenotype as fluctuations in the parameter lead to larger changes in phenotype.

Contrary to the previous model, in this case variation is not driven by differences in the neuromodulatory circuit, but rather the amplitude of neuromodulation alters the degree to which variability in other components of the nervous system manifests as idiosyncratic behavioral preferences (Figure 2). By changing the relationship between underlying variability in the circuit (Figure 2A) and either making the behavioral phenotype less sensitive to changes in the circuit parameter (Figure 2B) or more sensitive (Figure 2C), the variability in the population can be modulated despite no change in the underlying variability in the circuit parameter.

Clear experimental evidence of the ability of neuromodulators to modulate the manifestation of underlying circuit variability comes from the crustacean stomatogastric nervous system. The crustacean stomatogastric nervous system is divergent among individuals in terms of the constituent neurons (Bucher et al., 2007) and ion channels in individual neurons (Schulz et al., 2007). Variability in connectivity can be ameliorated through neuromodulation, as evidenced by a systematic search of synapse strengths leading to stereotyped rhythmic activity using dynamic clamp between pacemaker neurons (Grashow et al., 2009). Adding two neuromodulators, serotonin and oxotremorine, increased the underlying set of parameters that led to rhythmic bursting—in this way neuromodulators enable a larger distribution of underlying circuit parameters to produce similar behavioral output, increasing the robustness of the circuit.

Compelling experimental evidence for neuromodulation that increases population individuality by accentuating underlying network parameters is more difficult to find, though whether this is due to any evolutionary bias toward neuromodulators promoting robustness or researcher’s bias in studying robustness is unclear. Nonetheless, theoretical evidence in simplified models of neuronal circuits highlights that small shifts in conductances consistent with the method of action of neuromodulators change the sensitivity of the circuit to perturbations in other parameters (Goldman et al., 2001; Gutierrez et al., 2013).

A key insight from this work, as well as analogous classic work in evolutionary genetics (Rendel, 1962), is that any change in the mean of a phenotype will also change the variance in a phenotype if the shift in the mean is due to a shift in a parameter with a non-linear relationship to the phenotype, even if the variance in the parameter doesn’t change (Figure 2D). In this way, even neuromodulators with straightforward linear effects on one parameter may change the sensitivity of behavioral phenotype to other underlying circuit components, and hence affect the degree of individuality in a population. Similarly, the same neuromodulator may have differing effects based on the underlying state of the neural circuit (Figure 2E), leading to inconsistent or does dependent effects of neuromodulators on variability.

## Neuromodulation of Individuality Through Plasticity

Both previous categories assume that the manifestation of individuality is due to the acute influence of neuromodulators on the observed manifestation of individuality, however, some evidence suggests that even transient changes in neuromodulation in the animals past might drive changes in individuality. Application of the serotonin agonist ANW increases population variability even 5 days after washout (Kain et al., 2012), suggesting that neuromodulators may affect the development of individuality and have long lasting effects on the behavioral preferences of an animal. This is consistent with a large body of literature showing neuromodulators playing a critical role in gating plasticity and learning in a wide range of species (Damme et al., 2021), including *Aplysia* (Barbas et al., 2003), *Drosophila* (Kadow, 2019), mice (Diering et al., 2017), and humans (Likhtik and Johansen, 2019; Damme et al., 2021).

**FIGURE 3:**
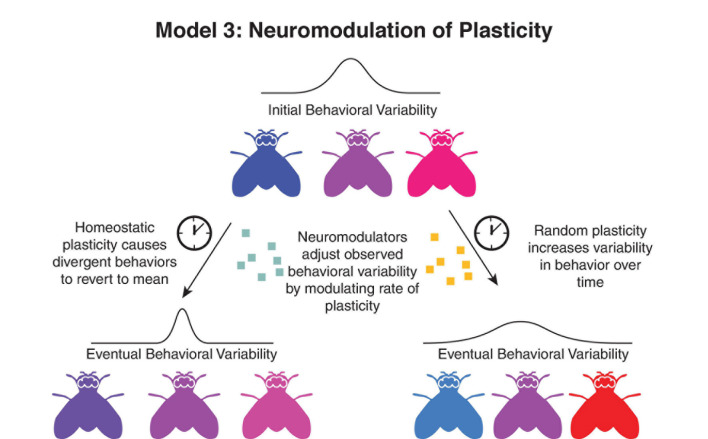
Neuromodulation as a regulator of variability through plasticity. In this model, variability is driven by changes in the variability of underlying circuit components over time, either in a homeostatic mechanism (left) moving behavior closer to a predetermined set point, or through a divergent method (right), leading to further deviation from parameters determined during development.

Neuromodulatory changes in learning could manifest in multiple directions (Figure 3). Neuromodulation could lead to the refinement of circuits, taking initially noisy developmental connections and applying a learning rule that drives them toward a more functional outcome. This is seen in *C. elegans*, where asymmetry in connections early in development is reduced as animals grow older (Witvliet et al., 2021). This sort of activity-dependent refinement of function, particularly during developmental critical periods has been demonstrated in a wide variety of systems and is influenced by neuromodulators (Shepard et al., 2015).

Alternatively, if development is more tightly controlled than plasticity, plasticity may drive further divergence of circuits over time. Estimations of the genetic heritability of personality traits decrease over time (Briley and Tucker-Drob, 2014), and even among animals raised under similar environments, cumulative changes in the circuit over time could lead to a greater array of idiosyncratic preferences. Even in cases with near perfect homeostatic learning rules, most changes in synaptic plasticity will be driven by spontaneous fluctuations (Raman and O’Leary, 2021), and misalignment between the homeostatic rules and the output behavior could lead to fluctuations in observed behavioral preference over time. This shift is supported by observations of idiosyncratic preferences—animals change their individual preferences over time, even in the absence of stimuli to induce learning (Buchanan et al., 2015; Werkhoven et al., 2021). In these cases, however, the overall distribution of preferences in the population remained constant over time—suggesting that either the divergent and convergent effects of plasticity in the circuit are balanced, or that changes in the range of preferences measured in a population operate via different mechanisms than those determining where in that range each individual occurs.

An additional possibility for neuromodulators to affect individuality through plasticity is by regulating other neuromodulators. Experimental manipulations of one neuromodulator can affect the strength of other neuromodulators (Niederkofler et al., 2015; Niens et al., 2017). Evidence suggests that these processes occur over long time scales, allowing shifts in one neuromodulator to rewire other neuromodulatory systems. Therefore manipulations of one neuromodulator could lead to changes in individuality via another neuromodulator using any of the mechanisms discussed in this paper. This possibility highlights the ways in which these different models can interact, and a given system might involve mechanisms that integrate elements from each of the three abstract models discussed in this paper.

## Future Directions

**FIGURE 4:**
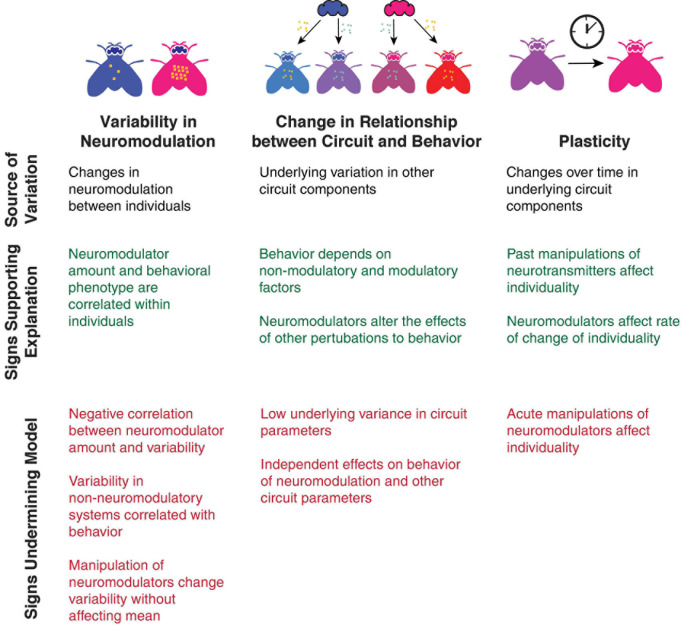
Tests for differentiating between models of how neuromodulators affect individuality. Observations that provide evidence for each model are listed in green, and observations that are contraindicative for each model are shown in red.

How then, does neuromodulation affect individuality? Despite suggestions from various studies, this remains an open question requiring more study. Nonetheless, a number of observations can help determine the answer to this question and categorize the role of neuromodulators in regulating individuality in particular behaviors (Figure 4):

• Does past neuromodulation and manipulation of neuromodulation affect individuality, or does neuromodulation alter the rate of change of individuality, suggesting an effect on plasticity?
• Do acute effects of neuromodulators on individuality wash out, suggesting they are reversible?
• Does neuromodulation alter the rate by which individuals change idiosyncratic preferences, suggesting that neuromodulators increase or decrease the rate of plasticity?
• Do individuals maintain idiosyncratic preferences when neuromodulatory systems are silenced, suggesting that individuality is not solely driven by neuromodulatory systems?
• Do neuromodulators have independent effects on the mean of a trait and its variability, suggesting that neuromodulators target specific processes regulating variability?
• Does increased neuromodulation increase or decrease the relationship between underlying circuit variability and behavioral phenotypes, suggesting a role in adjusting the gain of a trait?
• Do neuromodulators alter the effects of other perturbations of behavior, suggesting they are altering the role of other determinants of behavior?

These questions, based on hints from the current literature, will almost certainly lead to conflicting answers. The correlation in serotonin levels and dorsal raphe neuron physiology with behavioral variability suggests shifts in variability in neuromodulation is a part of the answer in the zebrafish acoustic startle response (Pantoja et al., 2016) but that same mechanism struggles to explain how serotonin deficient *C. elegans* demonstrate more variability in roaming (Stern et al., 2017). Neuromodulation increases robustness and decreases variance in behavior in crabs through acute changes in conductances (Grashow et al., 2009), but that doesn’t explain how a serotonin agonist can affect individuality 5 days after washout (Kain et al., 2012). These results suggest that none of these models are a universal solution, but instead, that neuromodulators may affect individuality via different mechanisms in different species, circuits and behaviors—and that each case may be a mix of multiple mechanisms.

Apart from the mechanism of action of neuromodulation on individuality, a number of other questions pertaining to neuromodulation and individuality remain unstudied. Does the amount of individuality in individuals change at different points in the lifecycle of an organism, and do these changes correlate to neuromodulator strength? Do changes in the environment or experience of an animal change the amount of individuality manifested in a population, and if so, is this controlled by neuromodulators? Understanding the mechanisms by which neuromodulations influence individuality and how they influence these questions will provide a more detailed understanding of the role and control of individuality in species.

## Conclusion

The study of variability among populations underlies a foundational question in biology: what principles are generalizable across all individuals and what features are idiosyncratic and optional. Understanding variability is key to understanding developmental and learning rules as well as cognitive and behavioral processes.

Neuromodulators appear to play a key role in regulating individuality in many behaviors. As outlined in this review, there are multiple methods by which they could do so—understanding how and why provides us an opportunity to understand the underlying process by which these behaviors develop. Are neuromodulatory systems inherently less stereotyped than other neural circuits, and is this difference a major driver in individuality? Or do neuromodulators reveal or conceal widespread variation amongst other components of the nervous system? To what degree are organisms born different, and to what degree do they grow to become different, even continuing into adulthood? Understanding how neuromodulators influence individuality will offer insights into broader questions about the mechanisms that create individuals.

## Author Contributions

RM conceived and wrote this review.

## Conflict of Interest

The author declares that the research was conducted in the absence of any commercial or financial relationships that could be construed as a potential conflict of interest.

## Publisher’s Note

All claims expressed in this article are solely those of the authors and do not necessarily represent those of their affiliated organizations, or those of the publisher, the editors and the reviewers. Any product that may be evaluated in this article, or claim that may be made by its manufacturer, is not guaranteed or endorsed by the publisher.
